# Knowledge Evaluation and Assessment of Bacterial Contamination of Toothbrushes

**DOI:** 10.1111/idh.70011

**Published:** 2025-12-04

**Authors:** A. A. L. Moura‐Filho, J. D. Bronzato, B. P. F. A. Gomes, C. H. F. Silva, S. L. Peralta

**Affiliations:** ^1^ Division of Endodontics, Department of Restorative Dentistry, Piracicaba Dental School State University of Campinas – UNICAMP Piracicaba São Paulo Brazil; ^2^ São João Calábria Dental Complex UniCatólica University Quixadá Ceará Brazil; ^3^ Department of Rehabilitative Stomatology Universidad Nacional Mayor de San Marcos Lima Peru

**Keywords:** bacteria, microbiology, oral hygiene, tooth‐brushing

## Abstract

**Objectives:**

This study aimed to quantify common microorganisms present in the toothbrushes of the participants and assess the level of their knowledge regarding this issue.

**Methods:**

Participants (*n* = 30) answered a questionnaire designed to elicit information which could be used to identify the habits regarding the use of toothbrushes and their storage. The participants were randomly selected and divided into two groups: Group 1 used a new toothbrush for 1 month and Group 2 used a new toothbrush for 3 months. Each participant was then instructed to store the toothbrush as usual and return it in a sterile bag. The toothbrush heads were cut for microbiological analyses. Cultivation media for the identification of *Streptococcus mutans* , *Staphylococcus aureus,* and Enterobacteriaceae were used and colony‐forming units were counted after the incubation period. Statistical analyses were performed by using a two‐way ANOVA test.

**Results:**

The majority of the participants had never received professional advice regarding the storage and disinfection of toothbrushes (93%) and they were unaware of the contamination degree of their toothbrushes (73%). It was not possible to verify a statistically significant difference between the 1‐month and 3‐month groups for *S. mutans* (*p* > 0.05). However, the evaluation of contamination by *S. aureus* and Enterobacteriaceae revealed statistically significant differences between these groups (*p* < 0.05).

**Conclusions:**

It was concluded that *S. mutans* , *S. aureus,* and Enterobacteriaceae were found in the toothbrushes analysed. However, the poor knowledge of the participants about the degree of contamination of their toothbrushes is a challenge to overcome, as most of them were unaware of the care required for toothbrush storage.

## 1 Introduction

The conditions in which the fetus develops contribute to the absence of microorganisms in the oral cavity, but after birth different microbial species begin to colonise the oral cavity. In fact, numerous authors have identified a diversity of microorganisms in the oral environment [1, 2]. Oral microflora, oral microbiota and human oral microbiome are terms used to describe the presence of eubacteria, archaea, fungi, mycoplasmas, protozoa and possibly significant amounts of viruses [1].

Oral hygiene habits are essential for establishing and maintaining oral health [3]. The toothbrush is the most efficient mechanical tool for oral hygiene as it disorganizes the dental biofilm and provides a healthy ecosystem [3]. Tooth brushing is the most widely disseminated, practiced and socially accepted method of oral hygiene, thus being consequently an essential practice for health promotion and prevention of dental diseases [4].

Important discussions have surrounded the possibility of microbial colonisation of toothbrushes and their subsequent infection. Toothbrushes can become contaminated upon the initial direct contact with oral structures, which results in the transfer of microorganisms from the oral microbiota to the toothbrush bristles [5]. Based on this assumption, toothbrushes end up acting as fomites as they become microorganism reservoirs by transmitting them or serving as sources of inoculation and reintroduction into the oral cavity [6]. Some of these organisms are identified as causing important infectious processes, such as ocular and urinary tract infections, osteomyelitis, bacterial endocarditis [7], arthritis, stroke, and even heart disease [8, 9].

Toothbrushes can be contaminated in the oral cavity, but a significant external influence also deserves attention, such as the storage environment, which may favour different microbial colonisation [10]. As a result, contaminated toothbrushes may introduce new microorganisms into the oral cavity [4]. In this context, the control of microbial contamination of toothbrushes has been primarily recommended as an effective method of disease prevention by means of their proper storage, disinfection, and replacement [6].

Although numerous scientific publications indicate that toothbrushes can be contaminated by a variety of microorganisms [10, 11, 12], there are still cases of a lack of awareness about the maintenance of toothbrushes, such as proper storage, replacement and disinfection [13, 14]. Therefore, this study aimed to quantify microorganisms commonly present in toothbrushes of the participants and assess the level of their knowledge regarding this issue, which contributes academically and socially to informing the population about the problem of toothbrush contamination.

## 2 Materials and Methods

### 2.1 Patient Selection

This cross‐sectional study was conducted according to the Strengthening of the Reporting of Observational Studies in Epidemiology (STROBE) statement [15].

The study was carried out at the São João Calábria Dental Complex and Microbiology Laboratory, both located at the UniCatólica University (Quixadá, CE, Brazil). The participants were informed about the objectives and procedures of the study before signing an informed consent form prepared in accordance with the guidelines of the Research Ethics Committee of UniCatólica under protocol number 1841036 and the National Health Council's Resolution 466/2012.

The sample consisted of a total of 30 individuals. The sample size of 15 participants per group was based on comparing the average of two independent groups in an unpaired quantitative assessment between the two sample sets [16]. The standard deviation values and the minimum difference used in the sample size calculation formula were based on a pilot study and are consistent with the results of this study.

Individuals aged between 18 and 60 years old who used a toothbrush as their primary method of oral hygiene and had no physical impairment met the inclusion criteria. Exclusion criteria were individuals on antibiotic therapy, who used antibiotics for at least 3 months prior to the study, who were undergoing orthodontic treatment or had an intraoral prosthesis, who were smokers, and those who were absent during any phase of the study. Also, participants who had any change in their toothbrush care habits during the study were excluded.

### 2.2 Sample Collection

All the participants answered the questionnaire (Table 1) designed to elicit information to be used to identify how their toothbrushes are used and stored. The participants were then randomly divided into two groups, in which 15 participants would use a new toothbrush for 1 month (Group 1) and 15 others would also use a new toothbrush for 3 months (Group 2). Another new toothbrush (Soft Pro‐adult Slide; Medfio, Curitiba, PR, Brazil) was microbiologically analysed and used as a control.

**TABLE 1:** Questionnaire applied to the participants of the study.

Questions	Responses	*n*	%
Gender	Male	12	40
	Female	8	60
Age	18–20 years old	4	13.33
	21–25 years old	6	20.00
	26–30 years old	12	40.00
	31–35 years old	5	16.66
	36–40 years old	3	10.00
	45–50 years old	0	0
	51–60 years old	0	0
Do you use a toothbrush as the main means of oral hygiene?	Yes	30	100
	No	0	0
Have you ever received instructions on storing and disinfecting toothbrushes?	Yes	2	6.66
	No	28	93.33
Are you aware of the degree of contamination of toothbrushes?	Yes	8	26.66
	No	22	73.33
How often do you change your toothbrush?	Once a month	3	10
	Every 2 months	10	33.33
	Every 3 months	11	36.66
	Every 3–4 months	6	20
	Every 5 months or more	0	0
After brushing your teeth, you	Do not wash the toothbrush head	0	0
	Wash the toothbrush head under running water	30	100
	Hit it against the sink to remove excess water from the bristles	10	33.33
	Run your finger over the bristles to remove excess water	20	66.66
	Dry the toothbrush head on a cloth towel	0	0
	Dry your toothbrush head with a paper towel	0	0
After using the toothbrush, do you use any type of antiseptic solution for the bristles?	Yes	1	3.33
	No	29	96.66
Do you leave your toothbrush stored inside the bathroom?	Yes	22	73.33
	No	8	26.66
If stored inside the bathroom, what condition is it in?	Lying on top of the bathroom sink	2	9.09
	On top of the sink, inside a container without a lid	6	27.27
	On top of the sink, inside a container with a lid	6	27.27
	Inside the bathroom cabinet	8	36.36
Do you store your toothbrush with other toothbrushes?	Yes	20	66.66
	No	10	33.33
Do you use a protective cover for your toothbrush bristles?	Yes	19	63.33
	No	11	36.66

Therefore, each participant received a new toothbrush and oral hygiene instructions, such as brushing three times per day and flossing. Next, they were instructed to store their toothbrush as usual and return it in a sterile bag provided by the researchers at a predetermined time. Additionally, the bag was used to transport the toothbrush to the microbiology laboratory. Before returning the contaminated brush, the participants were asked if there had been any change in their toothbrush care habits during this study time, that is, if they had taken any care measures with the toothbrush not taken previously.

After analysing the data, the participants were informed about the findings and given instructions on how to store their toothbrushes properly.

### 2.3 Microbiological Analysis

With regard to the sample preparation, the heads of each toothbrush were removed with a sterile cutting tool and placed in test tubes containing 10 mL of BHI liquid medium before being subjected to vigorous vortexing for 60 s. Next, serial decimal dilutions up to 10^−3^ were made and the diluted content was immediately seeded onto the culture medium. Different cultivation media were selected for analyses according to the target bacteria, namely: Mitis salivarius agar for *Streptococcus mutans* , MacConkey agar for enterobacteria, and mannitol salt agar for *Staphylococcus aureus* .

Petri dishes for *S. mutans* culture were placed in an anaerobic jar with a burning candle. After hermetically sealing the jar and extinguishing the candle (CO_2_ production), the whole set was incubated at 37°C for 48 h. The Petri dishes containing enterobacteria and *S. aureus* were kept at 37°C in a bacteriological incubator for 48 h. After this time, the colony‐forming units (CFU) were checked.

### 2.4 Data Analysis

Excel was used for data collection and tabulation, with Sigma Stat 3.5 software (Systat Software, CA, USA) being used for statistical analysis. The two‐way ANOVA test used a log_10_ transformation to assess the difference between the amounts of bacteria over time. To check if there was any association between the levels of bacteria and the answers to the questionnaire, the data were checked for normality by using the Shapiro–Wilk test and then analyzed through the Mann–Whitney test. A level of significance of 5% was adopted.

## 3 Results

**FIGURE 1:**
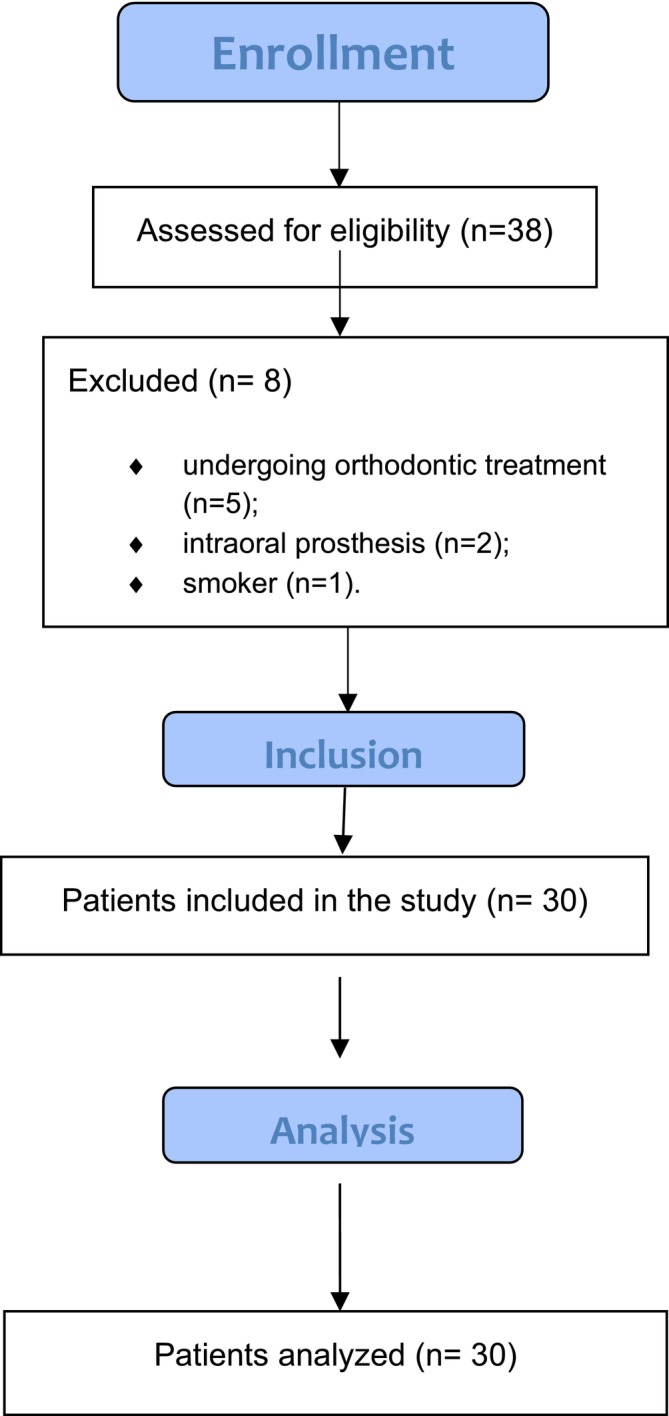
STROBE flow diagram. STROBE, Strengthening the Reporting of Observational Studies in Epidemiology.

The questionnaire was answered by 30 participants (Figure 1), of which 60% were female and 40% were male, aged between 18 and 35 years. The outcome of the questionnaire can be seen in Table 1.

By comparing Group 1 (use of toothbrush for 1 month) to Group 2 (use of toothbrush for 3 months), there was no statistically significant difference (*p* = 0.3) between the levels of *S. mutans* in toothbrushes (Table 2). However, data on *S. aureus* indicated that there was a statistical difference (*p* < 0.016) in the quantification of CFU/mL between both groups, with greater contamination in the final month (Table 2). Also, there was a statistically significant difference regarding Enterobacteriaceae, with higher formation of CFU/mL in the toothbrushes used for 3 months (*p* = 0.001) (Table 2).

**TABLE 2:** CFU/mL (mean ± standard deviation) in log_10_ of different microorganisms after 1 and 3 months of toothbrush use.

	1 month	3 months
*Streptococcus mutans*	A 4.2 ± 0.3 a	B 4.7 ± 0.3 a
*Staphylococcus aureus*	A 4.5 ± 0.3 b	AB 5.5 ± 0.4 a
Enterobacteriaceae	A 4.4 ± 0.3 b	A 6.0 ± 0.4 a

*Note:* Small letters compare lines, and capital letters compare columns.

By comparing the microorganisms after 1 month of using the toothbrushes, it was found that the microbial loads were similar. After 3 months of use, a greater number of enterobacteria, staphylococci, and streptococci were detected in this order (Table 2).

By associating the microbiological results with the questionnaire results, some significant differences were found, such as those in which Enterobacteriaceae (*p* = 0.00) and *S. aureus* (*p* = 0.002) counts were significantly higher in the group who did not hit their toothbrush against the sink. Indeed, the toothbrushes of participants who used their fingers to remove excess water from the bristles had significantly higher bacterial counts for Enterobacteriaceae (*p* = 0.000) and *S. aureus* (*p* = 0.002).

Storing the toothbrush in the bathroom was associated with significantly higher bacterial counts for Enterobacteriaceae (*p* = 0.002), *S. aureus* (*p* = 0.011), and *S. mutans* (*p* = 0.014). Storing the toothbrush with other ones also resulted in significantly higher bacterial counts for *S. aureus* (*p* = 0.017) and *S. mutans* (*p* = 0.041). However, the use of a toothbrush bristle cap was associated with significantly lower bacterial counts for Enterobacteriaceae (*p* = 0.002) and *S. aureus* (*p* = 0.039).

## 4 Discussion

Toothbrush contamination can be influenced by the storage environment. This observational study aimed to quantify common microorganisms present in toothbrushes and assess the level of people's knowledge regarding this issue by associating these two findings. The main findings revealed a significant lack of awareness and inadequate practices among the participants regarding toothbrush care, with many not receiving proper instructions on storage and disinfection, thus leading to toothbrush contamination.

Toothbrushes are significant reservoirs for environmental microorganisms, in addition to facilitating the transport and growth of microorganisms [17]. Toothbrushes are a potential source of oral reinfection by bacteria with a moderate degree of pathogenicity [18]. It was previously found that microbial transmission through toothbrushes of individuals with periodontal disease could result in transient bacteremia, which has the potential to compromise the systemic integrity of others [19]. These various organisms frequently spread as a result of trauma caused by toothbrushes. These impacts on oral structures provide an important systemic access for these microorganisms, allowing pathological processes to be established [20].

The bacterial genera and species described below were selected for this study due to their potential viability to develop in toothbrush bristles and their association with systemic infections. *Streptococcus* is the most common bacterial species in the mouth, with *S. mutans* being particularly abundant in caries lesions [21]. *Staphylococcus* spp. are highly adaptable organisms capable of tolerating both high and low temperatures, which include opportunistic species causing superficial and systemic infections [22]. *Staphylococcus* typically colonises the skin surface and is linked to communicating lesions in the oral cavity, such as a fistula [23, 24]. Species from the Enterobacteriaceae group have as their natural habitat the intestinal tract of humans and animals [25], with species such as *E. faecalis* being commonly found in dental infections [26].

This study found no statistical difference in the levels of *S. mutans* after 1 and 3 months of toothbrush use. By comparing data on *S. mutans* contamination to those found in the literature [27], it is possible to determine that the results are not comparable because, according to Raiyani et al., toothbrushes used for 3 months had higher levels of contamination by *S. mutans* than those used for 1 month. In contrast, another study found a greater proportion of toothbrushes contaminated with *S. mutans* in the 1‐month experimental group than in the 3‐month experimental group [7].

*S. mutans* has an excellent capacity to contaminate toothbrush bristles, as it is able to maintain viability for a short period of time, with studies identifying contamination within 15 days [28]. Besides being viable for a short period, constant tooth brushing may maintain the level of these bacteria in the toothbrush. This occurs due to the fact that Streptococci is the most prevalent species of bacteria in the mouth [29]. These commonly identified bacterial species are frequently associated with the onset of caries in humans, and their pathogenicity should always be judged primarily by their transmissibility potential [7].

*S. aureus* is implicated in significant infectious processes, including fever, anthrax, pustules, abscesses, osteomyelitis, endocarditis, and septicemia [30]. It is typically an opportunistic organism causing superficial lesions and systemic infections [30]. A study on the contamination of toothbrushes over a period of 1 month revealed that *S. aureus* was not present in toothbrushes kept in bathrooms without a toilet, whereas it was identified in environments with a toilet during the same period [7]. By comparing the microbial load of *S. aureus* at 1 and 3 months, the same author found that more toothbrushes were contaminated in the most recent month [7], which is in disagreement with our study that found higher levels of *S. aureus* after 3 months. This difference may be due to different storage and cleaning conditions of the toothbrushes between the studies. The presence of *S. aureus* is facilitated by its ability to colonise different environments by being able to present itself as a species contaminating the air, being present on different surfaces and having the capacity for rapid multiplication [30].

Considering the variable time, toothbrushes were more contaminated with Enterobacteriaceae during the 3‐month period, which is in accordance with another study [27]. In fact, the literature has highlighted greater amounts of *Klebsiella* and *E. coli* (CFU/mL) in 3‐month‐old toothbrushes contaminated by enteric bacteria [27], which are more prevalent in toothbrushes stored in bathrooms with a toilet [7]. Although not specifically identified in the present study, *E. coli* and *Klebsiella* are the most common Enterobacteriaceae found in toothbrushes [27]. Therefore, it is essential to note that *Klebsiella* and *E. coli* may be involved in the contamination when enteric bacteria are present.

Species belonging to the family Enterobacteriaceae are a highly relevant group and deserve consideration when identified as toothbrush contaminants. In addition to being regarded as indicators of unsanitary conditions, these bacteria are associated with a variety of diseases [31]. In addition to the inadequate storage of toothbrushes, aerosols from toilet flushing, tap water, hand hygiene (primarily fingers) and the humid environment of the bathroom all contribute to the spread of this type of contamination [32].

The findings of this study on storage and disinfection of toothbrushes corroborate those found in the literature [33], in which the majority of the participants reported they had not received any type of instruction regarding this issue. The present study also agrees with another one [34], which also showed this lack of awareness about the aforementioned problem, as the majority of the participants reported that they did not know the percentage of microbiological contamination in their toothbrushes or that they were even unaware of this fact.

The results showed that 37% of the participants changed their toothbrushes every 3 months, 33% every 2 months, 10% every month, and none after more than 5 months. In contrast, another study revealed that 27.3% of the respondents changed their toothbrushes every 2 months, 27.3% every 3 months, and 9.1% every 6 months [33]. This difference may be related to the population sample. Studies have demonstrated that different populations have different knowledge about the maintenance of toothbrushes. For example, dental students usually change their toothbrushes every month [14, 35], which may be because of their advanced knowledge in comparison to the general population.

As for how often toothbrushes should be replaced, the average recommendation is every 3 months [36]. However, this should not be an imposition as other factors have to be considered, particularly those related to physical destruction, which reduces the effectiveness of toothbrushes in cleaning oral structures [37]. Previous studies indicate that patients with systemic diseases, organ transplants or who are undergoing chemotherapy should replace their toothbrushes more frequently [38]. Individuals prone to infection, such as the elderly, transplant recipients and immunocompromised ones, should replace their toothbrushes every 2 weeks [18].

The strength of this study was to associate the bacterial contamination of toothbrushes with the participants' knowledge about toothbrush care. After tooth brushing, it is recommended that the toothbrush be thoroughly washed, especially the bristles and all excess moisture should be carefully removed. Our results corroborate the literature [33], in which most participants reported washing their toothbrushes after use. However, it was found that participants who used their fingers to remove excess water from the toothbrush bristles had significantly higher bacterial counts. According to other authors, the act of using the fingers to remove excess water is characterized as a potential source of contamination by *Staphylococcus* spp. [28, 39], which can also lead to contamination by *Pseudomonas* spp. and *E. coli* [40].

The initial step in the process of cleaning toothbrushes is to rinse them with water, which is the method most widely used by people [41]. However, the use of chemical solutions to eradicate large foci of microorganisms has evolved into a safe method. Previously, 84% of the dental students surveyed did not use antiseptic solutions as a disinfectant [35], indicating that this practice generated little interest. It is important to note that 97% of the participants in our study reported they did not use antimicrobial agents to decontaminate toothbrushes, which might have occurred due to a lack of knowledge.

The toothbrush should be stored after being cleaned. Storing the toothbrush in the bathroom was associated with significantly higher bacterial counts for all bacteria tested. Toothbrushes must be kept outside the bathroom, as other studies have shown that intensive colonisation by bacteria occurs, particularly, in this environment because it is highly contaminated [7, 27].

Storing the toothbrush with other ones also resulted in significantly higher bacterial counts, especially for *S. aureus* and *S. mutans* . This may be influenced if other toothbrushes are more contaminated than those used by the participants in this study. However, our study did not evaluate the contamination of other toothbrushes by the participants' family members.

The use of a toothbrush bristle cover was associated with significantly lower bacterial counts for Enterobacteriaceae and *S. aureus* . This may be due to the cover's protection against possible spread of microorganisms during toilet flushing as many of the participants stored their toothbrushes on the top of the sink in the bathroom [42]. Ideally, a holder allowing the toothbrush to remain vertical and ventilated in a place free of impurities is recommended to avoid microbial growth [10].

One of the primary limitations of a parallel study design used in the present work is the inherent risk that the two groups of participants, despite being randomly divided, might have intrinsic differences influencing the bacterial counts. Although randomization is a critical step in mitigating selection bias and ensuring baseline comparability between groups, it does not guarantee complete elimination of variability. Such intrinsic differences could include variations in oral hygiene habits, dietary patterns, genetic factors or pre‐existing oral microbiota compositions, all potentially confounding the results.

Future research is needed to address the limitations identified in this study. Additionally, focus should be on educating the general population about proper practices for toothbrush storage and disinfection and on evaluating the effectiveness of various disinfection methods.

## 5 Conclusion

*S. mutans* , *S. aureus* , and Enterobacteriaceae were found in the toothbrushes analysed. However, knowledge about the degree of contamination of toothbrushes is a challenge to overcome, as most of the participants were unaware of the basic care needed with toothbrushes.

## 6 Clinical Relevance

### 6.1 Scientific Rationale for the Study

The toothbrush helps to control bacterial biofilm on the teeth and periodontal surfaces. This oral hygiene practice, along with external factors such as toothbrush storage, causes contamination due to microbe retention and survival in the bristles, resulting in oral recontamination and systemic dissemination.

### 6.2 Principal Findings

The findings showed cases of poor awareness about toothbrush care, including a lack of recognition and use of proper storage, replacement and disinfection methods, and how this contributes to toothbrush contamination.

### 6.3 Practical Implications

This study provides academic and social justification for informing the population about the problem of toothbrush contamination.

## Author Contributions

All the authors conceived the ideas, collected the data, analysed the data, and wrote and reviewed the manuscript.

## Funding

This study was supported by the Sao Paulo Research Foundation (FAPESP grant numbers 2017/25090‐3 and 2021/13871‐6), National Council for Scientific and Technological Development (CNPq grant numbers 421801/2021‐2 and 303852/2019‐4), and Coordination for the Improvement of Higher Education Personnel (CAPES, finance code 001).

## Conflicts of Interest

The authors declare no conflicts of interest.

## Data Availability

The data that support the findings of this study are available from the corresponding author upon reasonable request.
